# Unraveling silence: police perceptions and responses to male victims of rape

**DOI:** 10.3389/fsoc.2026.1860214

**Published:** 2026-07-10

**Authors:** Katlego Vincent Scheepers, Letitia Smuts, Anthony Brown

**Affiliations:** 1 Department of Sociology, University of Johannesburg, Johannesburg, South Africa; 2 College of Graduate Studies, University of South Africa, Pretoria, South Africa

**Keywords:** hegemonic masculinity, heteronormativity, male rape, policing, saps, secondary victimization

## Abstract

**Introduction:**

Male victims of rape face significant barriers when reporting to the South African Police Service (SAPS), where hegemonic masculinity and heteronormative institutional cultures render male victimhood socially unintelligible. This study examines how police perceptions and institutional practices produce epistemic and secondary victimization for male rape victims.

**Methods:**

A qualitative design using in-depth semi-structured interviews was employed. Fourteen SAPS officers in Johannesburg were recruited via purposive and snowball sampling. Data were analyzed using thematic content analysis following Braun and Clarke's six-phase framework, within an interpretive-constructivist epistemology, an intersectional theoretical framework, and Connell's hegemonic masculinity theory.

**Findings:**

Key themes emerged: (1) internalized rape myths and stigma as barriers to reporting male rape; (2) heteronormativity, homophobia and policing of male victimhood homophobia and the role of heteronormativity and homophobia in policing male victimhood. The section also addresses secondary and epistemic victimization through dismissive and intrusive questioning, and emergent pockets of resistance among officers advocating for more inclusive, victim-centred practices.

**Discussion:**

The findings demonstrate that hegemonic masculinity functions not only as a social ideology but as an institutional logic governing recognition, credibility, and access to justice for male rape victims. Gay and gender-nonconforming individuals face compounded stigma. Structural interventions, including gender-sensitive training and institutional reform, are needed to address the systemic marginalization of male rape victims within South African policing.

## 1 Introduction

This paper contends that the South African Police Service (SAPS) functions as one of the key institutional sites in the reproduction of hegemonic masculinity, where male rape is frequently constructed as implausible. In this context, police responses are not neutral but are shaped by deeply embedded gendered norms and heteronormative assumptions that render male victimhood socially unintelligible. As a result, male victims of rape encounter the trauma of rape, but also forms of secondary victimization within the system tasked with delivering justice.

In South Africa, the reporting of rape cases remains a challenge, particularly among male victims. Research by Lehner (2017) asserts that this contributes to the under-reporting of rape crimes to the police. For example, research by Mgolozeli and Duma (2020a) reveals that males are often subjected to long waiting periods at the South African Police Service (SAPS) reception before the case file is opened, tardy responses of police personnel in proceeding to examine the crime scene where the victim was sexually assaulted, victim-blaming, and the police discouraging victims from laying charges. Moreover, male victims are often more ferociously questioned by the police to verify incidents of their violation (Mgolozeli and Duma, 2020a; Patterson, 2011), which shows the dehumanizing and gender identity character assassination of males who report being raped to the police. More research found that the sexual orientation of male-to-male victims of rape often comes into question, with doubts around rape also emerging due to the males developing an erection during the incident, while there is a tendency for female-to-male rape incidents to be disbelieved (Masilo and Davhana-Maselesele, 2016; Naidoo, 2013). The responses reflect a broader societal construction of masculinity that is also institutionalized within policing practices, shaping how cases are received. In South Africa, this institutional logic cannot be disentangled from race, the racialised history of the country, and specifically the colonial and apartheid-era construction of Black male sexuality as hypersexual and threatening, has produced particular assumptions about which male bodies can be rendered credible victims (Ratele, 2014; Gqola, 2015). These racialised scripts sit alongside gender norms within the very institution tasked with responding to sexual violence. As a result, distressing experiences reported with the police in South Africa have led to distrust and have discouraged the male victims of rape from seeking justice (Johnson et al., 2017; Jamel, 2014).

The police service constitutes an important societal setting in which gender becomes institutionalized. Normative ways in which masculinity and femininity are constructed and signified can be seen within the police, amongst both men and women officers. How police officers negotiate notions of masculinity is also evident when they interact with male victims of rape.

While there has been research on the marginalization of male victims shown above, less attention has been paid to how police officers reproduce these exclusions through everyday practices and interactions in South Africa. Drawing on qualitative interviews with SAPS officers, the study examined how cultural beliefs, organizational norms and individual perceptions intersect to shape police responses to male victims of rape. By foregrounding these narratives, the paper demonstrates how hegemonic masculinity operates not only as a social ideology but as an institutional logic that governs recognition, credibility, and access to justice.

As previously expressed by Messerschmidt (1993), the police have access to both power and resources, often positioned within hegemonic masculinities—a socially accepted form of masculinity that legitimizes men's power and dominance, and refer to expected ideals and norms associated with being a “real man”, such as toughness, heterosexuality and emotional constraint (Connell, 1995; Maake and Smuts, 2024). To understand how male victims of rape are treated in comparison to female victims of rape by the SAPS, it was essential to explore and understand how culture and history inform the multiple identities that may influence views on masculinity. In this context, it is also important to distinguish between sex, which refers to biological characteristics, and gender, which refers to the socially and culturally constructed roles, behaviors, and expectations associated with being male, female, or gender diverse. This also filled the gap in understanding and addressing the secondary victimization that male victims of rape experience when they engage with the police. Thus, analyzing police perceptions toward and responses to male victims allows for the recognition and comprehension of the challenges that these victims face, regardless of sexual orientation [or identity].

## 2 Literature review

Previously, Lees (1997) argued that when society and the police hold misguided views such as “men cannot be raped”, or that “male rape is a gay problem” or “they asked for it”, police responses to the victims become problematic. Men who have experienced rape often do not report their victimization due to fear of stigmatization and not being believed. Discriminatory attitudes and responses from police officers may leave male victims of rape feeling emasculated, ashamed, humiliated, and embarrassed (Abdullah-Khan, 2008). When society and police officers accept, and label men as sole perpetrators rather than victims, their experiences may be excluded or minimized through police attitudes and responses (Javaid, 2015b; Becker, 1963). The disconnect between police occupational culture and the broader cultural consciousness regarding male victims of rape reflect the persistence of patriarchal gender oppression. Therefore, it was crucial to explore police officers' subjectivities when handling rape cases, particularly given the sociocultural factors shaping their views on male victims of rape. In the quest to recognize an adequate ethical framework for police practice, Rowe (2008) notes that the process is both excessively and frequently influenced by cultures that promote stereotypes and discrimination within police officers' attitudes.

It was also essential to explore how hegemonic masculinity influences perceptions of male victims of rape and informs institutionalized sociocultural practices within policing. As mentioned earlier, Connell (1995) argues that hegemonic masculinity places men in a dominant societal position, characterized by strength and assertiveness. From early childhood, men are taught to hide their physical and emotional vulnerability and are socialized to identify themselves as powerful, forceful, and able to protect themselves and others (Singh, 2005; Kassing et al., 2005). This gender hierarchy also privileges heterosexuality and places any man who falls outside of this category in a minority position. Consequently, male victims of rape may be viewed as incompatible with dominant ideals of masculinity because vulnerability contradicts expectations that men should be sexually dominant and not be vulnerable (King, 1992; Singh, 2005).

Historically, hegemonic masculinity has been culturally glorified as the ideal form of masculinity associated with power and dominance (Messerschmidt, 2012). (Connell 1990, p. 94), further argues that the valorisation of hegemonic masculinity leads to stability in its perpetuation, reaffirming its “structure of dominance and oppression in the gender order as a whole”. Research across southern Africa demonstrates how hegemonic masculinity is tied to aggression, toughness, physical strength, and control. For example, according to Morojele's (2011) and Jamel's (2010) research, specific characteristics of hegemonic constructions in Lesotho include aggressiveness, roughness, physical strength, and an indifferent attitude. Mamba's (1997) paper also expresses that boys in Eswatini are socialized to exhibit elephant-like strength since it is their ultimate responsibility to be victorious warriors who can continue the tradition started by the early king Mswati. The norm of power and resistance is where Ratele (2008) and Langa (2020) place the hegemonic construct of young Black masculinities in South Africa. This indicates that, in relational circumstances, including for both men and women, having characteristics such as toughness, control, and hypersexuality is linked to hegemonic maleness.

Among the police, Reiner (2010) points out that police officers often enact machismo, an element of a male-dominated police subculture, which coincides with traditional constructs of hegemonic masculinity. The reactions that Reiner is pointing toward should also be seen within a broader context of South Africa's history of violence, which has developed a toxic masculinity that thrives on the political spectrum (Gqola, 2015). Turchik and Edwards (2012) and Loftus (2008) point out that masculine values that promote male power, invulnerability, and heterosexuality, contribute toward the creation and widespread acceptance of rape myths that shape how male victims of rape are both acknowledged and perceived in society. This includes the idea that only gay men are raped. For this research, it was therefore essential to explore what SAPS officers think are the circumstances that contribute to the rape of a man.

Male rape myths consistently undermine the seriousness of sexual violence against men, shifting responsibility away from perpetrators and toward victims. Davies and Rogers (2006) argue that dominant societal ideas about masculinity create a particular kind of shame for male survivors, one that discourages reporting and, by extension, limits the research and care available to them. This silence is not accidental. As (Graham 2006, p. 189) notes, the scarcity of literature on male sexual violence does not reflect the rarity of the problem but rather tells us something about the social conditions under which certain experiences are permitted to count as serious harm.

This is, at its core, an epistemic problem. When society fails to recognize male rape as a widespread and legitimate form of victimization, it produces a knowledge gap that leaves male victims without frameworks for understanding their own experiences. Abdullah-Khan (2008) points to deeply embedded sociocultural myths as a key reason why male rape continues to be overlooked at a global level, not simply as a policy failure, but as a fundamental gap in collective understanding.

The consequences, however, extend beyond what society knows or believes. Once a male rape victim does come forward, he faces a second layer of victimization rooted in how institutions respond to him. Reconciling the experience of victimization with prevailing heteronormative ideals of masculinity is not only a personal struggle; it shapes how police and other authorities perceive and treat male victims, opening the door to secondary victimization (Maier, 2008). Those who internalize rape myths are more inclined to attribute blame to victims rather than perpetrators (King and Hanrahan, 2015), and a consistent body of research suggests that male victims face greater blame than their female counterparts (Sleath and Bull, 2010; Chapleau et al., 2008; Davies and Rogers, 2006; Struckman-Johnson and Struckman-Johnson, 1994; Ostermann and Watson, 2024; PeConga et al., 2022). It is therefore unsurprising that underreporting remains strongly associated with the acceptance of male rape myths (Bullock and Beckson, 2011; Varga, 2001), as victims have little reason to expect that coming forward will lead to understanding rather than further harm.

Cultural, religious, social, and emotional barriers prevent male victims of rape from reporting their rape to the police and receiving the help they require (Badenoch, 2015). This is problematic for a variety of reasons. For one, these victims may be lonely and mentally scarred by their ordeal. Another effect is that cultures may continue to ignore the occurrence of male rape, either neglecting or overlooking it and allowing gender norms and beliefs to remain unquestioned. It was thus important to ask police officers how they think stigmatizing male rape may lead to trauma for male victims of rape.

As previously stated, social discourses often suggest that the experience of male rape “belongs” to members of the gay community, where it is common for heterosexual males and females to believe that only gay men can be raped (Rumney, 2009; Kassing et al., 2005). Javaid (2017) states that gay male victims of rape modify and contest hegemonic masculinity. As a result, male rape is perceived as a gay problem. Another reason gay males may be targets of perpetrators is the effort to undo the non-normative male sexual orientation. In South Africa, this crime is often referred to as “corrective rape”. As such, when gay men are raped, it is likely that those who live by the principles of hegemonic masculinity will support this homophobic attack and not regard it as a crime but instead perceive it as a corrective to gay male deviance. Importantly, corrective rape in South Africa is not a racially neutral phenomenon; it occurs predominantly in Black, economically marginalized communities, where the intersection of race, poverty, township geography, and patriarchal gender norms compounds both the risk of victimization and the inadequacy of institutional response (Gqola, 2015; Reid, 2018). For example, South Africa witnessed the gruesome murder and alleged rape of Lonwabo Nolte Jack in April of 2021, which made headlines, and shone a light on how hate crimes toward gay men continue unabated in contemporary South Africa (Ntseku, 2021). This is not the only murder and rape gay males continue to endure in South Africa, which forces most of them to live in fear.

Furthermore, certain religions fail to account for same-sex attraction, classifying gay men as “abnormal,” “deviant,” or “effeminate”. This is also the case in many African societies where these religions can be found. The concept of the macho male, the male wielding power, is reserved for heterosexual men, leaving gay men socially marginalized and isolated (Ferrales et al., 2016; Connell, 2005). Male rape is crucial to the question of sexuality, where, according to Walker et al. (2005), homophobia negatively influences views toward male rape and, worryingly, the processing of male rape cases within the justice system.

To the extent that heterosexuality and gender binarism remain institutionalized within society, they may undermine the quality of services provided to male rape victims by police. Javaid (2015a) argues that male victims' bodies are governed through social and power relations enacted by police. Research across South Africa, England, the United States, China, Australia, and Nigeria similarly demonstrates that police responses to male rape victims are often inadequate and dehumanizing (Abdullah-Khan, 2008; Jina et al., 2020; Mgolozeli and Duma, 2019, 2020a,b; Olatunji, 2012; Rumney, 2008a; Westmarland and Gangoli, 2011). Loftus (2010) and Olatunji (2012) further argue that police culture is often shaped by skepticism and conservatism, meaning that male rape victims may be constructed as the “other” because rape is still widely perceived as something that only happens to women. It was therefore important to explore the institutional cultures shaping police responses to male rape, including whether male and female officers respond differently to victims.

## 3 Theoretical framework

The study applied an intersectional framework to understand the intersecting identities related to race, gender, class, sexuality, and other characteristics that shape police responses toward male victims of rape (Crenshaw, 1989). An intersectional lens in this study exposes how police officers' perceptions toward male rape emerge from personal social locations, belief systems privileging certain masculinities, and institutionalized biases legitimizing particular victim profiles. It illuminates how stereotypes shaping the police officer's responses are constituted at the nexus of masculinity politics, cultural ideologies of sexuality, and gender performance norms. Crucially, race is not merely one axis among several in this context; in South Africa, it is a structuring force that has historically determined whose experiences of violence are made visible and whose are systematically overlooked. The construction of Black masculinity under colonialism and apartheid, as simultaneously threatening and sexually excessive, produced a racial grammar that continues to shape how Black male victims are read within institutions, including the police (Morrell et al., 2012; Ratele, 2014). An intersectional analysis, therefore, requires attending to how race, gender, and sexuality co-produce the specific forms of disbelief and dismissal that male rape victims encounter when they approach the SAPS. This theory captures the distinctive marginalization contours shaping gay, transgender, disabled, or socioeconomically disadvantaged male victims' experiences interfacing with a masculinised policing institution (Javaid, 2018). Intersectionality is used as a framework for understanding power asymmetries, which in this context helped in understanding how both men and the responses that SAPS officers provide to them reveal the relationships of inequality.

However, intersectionality, as a theoretical and analytical framework, operates not only at the individual and interactional/institutional levels that the findings from this study most directly address, but also at the level of intersecting systems of oppression (Crenshaw, 1989; Morrell et al., 2012). Patriarchy, heteronormativity, and the racial orders produced through colonialism and apartheid are not merely contextual frameworks but enduring structural systems that shape dispositions and sustain the institutional practices through which the findings documented here are produced. The responses of police officers toward male victims of rape and the individual beliefs underlying these responses are perhaps more usefully viewed as symptomatic rather than as isolated failures of training or personal bias. While a full structural analysis falls outside the scope of what the findings in this study can sustain, acknowledging this systemic dimension is essential to an intersectional reading of the findings.

The study also used Connell's theory of hegemonic masculinity regarding police officers' perceptions and responses, which relegate and subordinate male victims of rape for not achieving or living up to the principles of hegemonic masculinity. Hegemonic masculinity theory conceptualizes masculinities as socially constructed gender configurations operating within a hierarchical system privileging a dominant, institutionally empowered ideal (Connell, 2005; Connell and Messerschmidt, 2005; Oyewùmí, 1997). This hegemonic masculinity is defined by its ability to subordinate femininities as inherently inferior and relegate alternative masculinities like gay, disabled, or working-class masculinities to marginalized statuses (Jewkes et al., 2015). The hegemonic ideal maintains cultural ascendancy through its institutionalization in the arenas such as the government and religion. In the South African policing context specifically, this institutionalization carries a racial dimension that cannot be separated from gender; the apartheid-era police was constructed around a particular racialised and heteronormative masculinity, and while post-apartheid transformation has altered its demographic composition, it has not fully dismantled the cultural scripts that govern how victimhood is recognized and responded to within the institution (Marks, 2005; Newham et al., 2006).

As men deal with emasculation and humiliation in displaced circumstances, as well as the reality of oppositional gender relations, some scholars advise on the applicability of applying the hegemonic relational approach. This is to fully understand the interpersonal violence associated with masculinities in terms of how it expresses itself as vulnerable and powerless concerning women within transforming gender societies (Ratele, 2008). Zungu (2025) observes and discusses the variations in gender systems and dynamics in defining and constructing masculinities.

These theories complement each other and contribute to the sociology of policing and gender by reconceptualising hegemonic masculinity not merely as a cultural framework but as institutional logic that structures recognition, credibility, and response. The perspectives yielded invaluable insight into the ways hegemonic masculine politics are sustained and contested within the police (service), how lived realities of gender oppression are experienced and negotiated across marginalisations of race, class, sexuality, disability, and other salient identity axes, and how integrating more inclusive counter-scripts around masculinity and victimhood may ignite organizational transformation.

## 4 Methodology

This study, which forms part of the first author's PhD thesis, employed a qualitative research design using in-depth semi-structured interviews to explore SAPS officers' perceptions and responses regarding male rape victimization. The research is grounded in an interpretive-constructivist epistemology, which recognizes that social realities are constructed through human interactions, language, and institutional practices (Creswell and Poth, 2018). This philosophical orientation was particularly appropriate given the study's aim to understand how police officers construct meanings around male rape victimization and how these meaning-making processes shape institutional responses.

Qualitative methodology was selected for several compelling reasons. First, the exploratory nature of the research questions required an approach capable of capturing nuanced, context-specific understandings that quantitative methods would miss (Patton, 2015). Given the paucity of existing research on SAPS responses to male rape victims, a qualitative approach allowed for a discovery of unexpected themes and patterns. Second, the sensitivity of the topic necessitated face-to-face engagement where trust could be established and officers could articulate complex, potentially contradictory perspectives in their own words. Third, qualitative interviews enabled an exploration of officers' subjective meaning-making processes, revealing how institutional cultures, personal experiences, and broader gender ideologies intersect to shape responses to male victims (Denzin and Lincoln, 2018).

The choice of semi-structured interviews specifically balanced the need for systematic data collection across participants with flexibility to pursue emergent themes unique to individual police officers' experiences. This format allowed the researcher to ensure core topics were addressed while remaining responsive to participants' expertise and insights (Kvale and Brinkmann, 2015).

Participants were recruited through a combination of purposive and snowball sampling techniques, appropriate given the specialized population and sensitivity of the research topic (Noy, 2008). Purposive sampling allowed deliberate selection of officers with relevant experience in sexual violence case handling, while snowball sampling enabled access to officers who might not have responded to formal recruitment channels but were recommended by colleagues as having valuable perspectives.

Inclusion criteria specified that participants must be: (1) active SAPS officers in Johannesburg stations; (2) have at least 5 years of service experience - to ensure informed perceptions from officers who have had sufficient exposure to policing practices, institutional culture, and sexual violence cases within the SAPS; (3) have encountered or processed sexual violence cases in their duties; and (4) willing to discuss their perceptions of male rape victimization. Officers from various ranks and specializations were sought to capture diverse institutional perspectives, from frontline constables who typically conduct initial victim interviews to detectives.

Initial recruitment involved approaching station commanders at Johannesburg police stations, explaining the research purpose, and requesting permission to invite officers to participate. Station commanders/senior personnel identified potential participants meeting the inclusion criteria and facilitated introductions. Subsequently, early participants recommended colleagues whom they believed had relevant experiences or strong perspectives on the topic, generating additional participants through snowball sampling (Biernacki and Waldorf, 1981).

The final sample comprised 14 SAPS officers across varying ranks and experience levels. The sample included 10 male and 4 female police officers, all identifying as Black South Africans, reflecting the demographic composition of operational-level policing. Participants' years of service ranged from 5 to 15 years, and ranks included constables, sergeants, warrant officers, and a captain. The absence of White and Indian participants reflects the demographic reality of SAPS at operational levels, shaped by post-apartheid employment equity policies and the historical exclusion of Black South Africans from policing careers during apartheid (Newham et al., 2006). This demographic composition, while limiting transferability to other contexts, strengthened the study's ability to examine how officers navigating intersections of race, class mobility through police employment, and masculinity perceive male rape victimization. The racial homogeneity of the sample is therefore not merely a demographic footnote but a substantive analytical consideration. The data speaks to how Black officers, navigating their own racialised and classed positions within a post-apartheid institution, construct and respond to male victimhood. Race, in this sense, is not background context but an active part of how meaning is made within the SAPS, and it is treated as such in the analysis that follows.

Data collection occurred between November 2023 and May 2024 through semi-structured interviews. The interview guide was developed through iterative consultation with the first author's research supervisors. The guide addressed five thematic areas: (1) general perceptions of sexual violence and rape; (2) specific understandings of male rape victimization; (3) training and institutional preparedness for male cases; (4) experiences with male victims who had reported; and (5) recommendations for improving institutional responses.

Interviews averaged 55 min in duration (range: 35–85 min). Ten interviews were conducted face-to-face at neutral locations (private rooms at police stations, per participants' preference), while four were conducted by phone at participants' request due to scheduling constraints. Face-to-face interviews were prioritized for their advantages in rapport-building and observing non-verbal communication, but telephonic interviews proved equally rich when participants felt comfortable with the first author (Novick, 2008).

At the start of each interview, participants received detailed information sheets explaining the research purpose, the voluntary nature of participation, confidentiality protections, and the right to withdraw. Written informed consent was obtained for face-to-face interviews; verbal recorded consent was obtained for telephonic interviews. Participants were assured their identities would be protected through pseudonyms and that no identifying information would appear in publications or be shared with SAPS management.

Given the sensitive nature of discussing sexual violence, the first author employed several strategies to create safe interview spaces. These included: beginning with less threatening general questions before moving to specific topics; explicitly acknowledging the difficulty of the subject matter; offering breaks when participants appeared uncomfortable; and closing interviews by asking participants how they felt about the discussion and providing counseling referral information (Dickson-Swift et al., 2009). Interviews were conducted with sensitivity to emotional responses, and participants were given control over pacing and topic depth.

All interviews were audio-recorded with permission and transcribed verbatim by the first author. Transcripts were checked for accuracy against recordings, and pseudonyms were assigned during transcription to protect confidentiality. Field notes documenting non-verbal observations, interview context, and reflections were maintained separately.

Thematic content analysis was employed to analyse interview transcripts, following the six-phase framework outlined by (Braun and Clarke 2006, p. 87): (1) familiarization with data; (2) generating initial codes; (3) searching for themes; (4) reviewing themes; (5) defining and naming themes; and (6) producing the report.

The analysis process began with repeated reading of all transcripts to achieve immersion in the data. Initial codes were generated systematically across the entire dataset to identify features relevant to the research questions. This coding was both data-driven (inductive) and theory-driven (deductive), as the authors remained open to unexpected patterns while also sensitized by hegemonic masculinity and intersectionality theoretical frameworks (Fereday and Muir-Cochrane, 2006).

Codes were collated into themes representing patterned meanings across the dataset. These preliminary themes were reviewed against coded extracts and the entire dataset to ensure they accurately captured data patterns and addressed research questions. Themes were refined through iterative review, with some merged, split, or discarded as analysis progressed. The final thematic structure comprised four major themes, each with sub-themes capturing distinct dimensions of officers' perceptions and experiences.

Analysis was conducted manually using Microsoft Word documents for coding and theme organization, supplemented by Excel spreadsheets to track codes across participants and demographic variables. While qualitative analysis software offers advantages, manual analysis enabled intimate engagement with data and transparent audit trails (Basit, 2003).

The interviews were not conducted exclusively in English. Given the linguistic diversity of South Africa, participants were encouraged to speak in whichever language they felt most comfortable expressing themselves, to avoid the risk of flattening nuance and distorting meaning. All interviews were conducted by the first author, who is fluent in speaking, writing and understanding the languages participants expressed themselves in. This multilingual fluency allowed participants to articulate their experiences in the full depth and texture of their home languages without the mediation of an interpreter.

Where participants responded in languages other than English, the first author transcribed and engaged with the data directly in those languages before writing up the findings. Excerpts that appear in languages other than English have been captured as is, preserving the original phrasing and expression of participants. This decision was deliberate. Translation, even when careful, carries the risk of substituting the researcher's interpretation for the participants' intended meaning. By retaining the original language in the excerpts presented, we acknowledge the essence of what was communicated and allow the participant's voice to remain intact, rather than filtered through an additional layer of interpretation. Where necessary, contextual explanations are provided to assist readers who may not be familiar with a given language, without compromising the authenticity of the original account (Creswell, 2013; Ngozwana, 2018; Ntuli and Mudau, 2025).

The research received ethical approval from [institution removed for blind review] Humanities Ethics Committee (Reference: [removed]) and institutional permission from SAPS National Head Office. Multiple ethical considerations, such as informed consent, confidentiality, minimizing harm, and power dynamics, were addressed.

Participants received comprehensive information about research aims, procedures, risks, and benefits before consenting. Consent was treated as ongoing, with participants reminded of their right to withdraw at any point. Participants' identities were protected using pseudonyms, any identifying details were removed from the transcripts, and the data was stored securely on a password-protected device accessible only to the researcher and supervisors. No information linking participants to their statements was shared with the SAPS management.

Throughout the study, we maintained a reflexive stance, remaining aware that our own positionality, assumptions, and interactions shape data collection and analysis (Berger, 2015). We acknowledge that knowledge is always situated, making transparency about our social locations particularly important in interpretive research.

The first author, who conducted all interviews and led the data analysis, identifies as a Black South African gay man. This positioning offered meaningful points of connection with the study's concerns as someone who moves through the same racialised, gendered, and heteronormative institutional landscapes that shape the experiences explored. He brought both proximity to the subject matter and an embodied understanding of the stakes involved in how male victimhood is constructed and dismissed. At the same time, he remained critically aware of the need to ground interpretations in participants' accounts rather than personal experience, using a reflexive journal throughout data collection and analysis to interrogate these assumptions.

The second author identifies as a White South African heterosexual woman. As an outsider to the primary community under study, she contributed alternative interpretive perspectives and critical distance, particularly regarding the intersections of race, gender, and constructions of masculinity within SAPS. Her engagement with feminist scholarship on gender and institutional power also informed the study's theoretical framing.

The third author identifies as a Black South African gay man. His contribution enriched the analysis by further interrogating how race, sexuality, and masculinity intersect to shape experiences of stigma and secondary victimization. Regular meetings, supervision, and peer debriefing (Denzin and Lincoln, 2018) enabled us to critically examine our interpretations and ensure that our collective positionalities strengthened, rather than constrained, the analytical process.

Trustworthiness was ensured by applying Lincoln and Guba's (1981) work. Credibility was established through prolonged engagement with participants and triangulation across diverse perspectives. Transferability was supported by providing a thick description of the context, participants, and findings, enabling readers to assess its applicability to other settings. Dependability and confirmability were achieved through transparent documentation of the research process and reflexive practice.

Several methodological limitations warrant acknowledgment. First, the sample's demographic homogeneity (all Black officers from Johannesburg) limits transferability to other racial groups or geographic contexts within South Africa. Second, social desirability bias may have influenced officers to present themselves or SAPS favorably, though the candid critiques many offered suggest this was minimized. Third, while saturation was achieved for the primary research questions, additional interviews might have revealed further nuances. Fourth, the study captures officers' reported perceptions but not their actual behaviors when encountering male victims, highlighting the distinction between espoused attitudes and enacted practices (Argyris and Schön, 1974). Despite these limitations, the study's methodological rigor and theoretical grounding, along with the rich, contextually situated findings that were generated make significant contributions to understanding police responses to male rape victimization in the South African context.

## 5 Findings: societal and institutional cultures, secondary victimization, and police officers' responses to male rape

This section explores how societal norms, institutional cultures, and hegemonic constructions of masculinity shape police officers' perceptions and responses to male rape victims within the SAPS. The findings highlight how stigma, heteronormativity, and homophobia contribute to the marginalization and secondary victimization of male rape victims, while also demonstrating how some officers challenge these attitudes through more inclusive policing practices. Drawing on the concept of epistemic victimization, the section further examines how male victims are often denied recognition as credible knowers of their own experiences within dominant understandings of rape and victimhood.

### 5.1 Internalized myths and stigmas as barriers to reporting male rape

A prominent theme was the persistence of male rape myths and stigma as barriers to reporting and quality police response. Several participants noted the societal belief that men cannot be raped, framing male victimhood as emasculating and shameful. Lebogang and Oratile explained that many men fear reporting sexual victimization due to the stigma surrounding male rape and the widespread perception that they will be mocked or ridiculed at police stations. They shared:

“*I know there's this thing of saying. Yeah. If you are a man, you go to a police station, you say a woman is abusing you. They are laughing at you… I've been in this police station. I've been a police officer for 15 years. I have never in this police station. Unless if it's happening at another police station where a victim or a male comes in to report domestic violence, and then they are laughed at. I can for my police station where I am working now, I can attest to that. People are just assuming that, oh, I'm a man, if I go and report, the police are going to laugh at me, maybe it happened a while back, I don't know, but I've never witnessed it. I've never heard of it here*.” (Lebogang, Police officer)

“*What I heard is that most of them, they say they are scared to be laughed at… I have never seen it. I just hear people saying that. How can you go to the police station as a man to report? Maybe you go to report and the police will laugh at you. But I've never experienced it. Or maybe it's what they are assuming is happening… Here, it cannot happen. Maybe in the townships*.” (Oratile, Police officer)

This denial by relocation reveals that the institutional laughter is acknowledged as real but placed elsewhere, “maybe in the townships”. This preserves the officer's self-image while confirming the widespread fear that shapes victim behavior. Tebogo also echoed this, sharing that while he has not personally witnessed a male victim being mocked, he has “heard members of the gay community saying that you guys [police officers] laugh at us and don't take our cases forward.” Drawing on Mbembe (2001), laughter wielded by authority figures becomes a mechanism of oppression and silencing. It is a bonding mechanism that reinforces group cohesion at the expense of victims and may also function as a coping mechanism for officers ill-equipped to deal with male rape cases.

Abel added in Sepedi that when reporting, some police officers may say, “Hao bago rape jwang o le monna” [How do they rape you if you are a man]. Abel's remark captures how the police station can become a site of epistemic victimization, where the very act of reporting is met not with belief but with a question that functions as a dismissal. The phrase reflects a deeply embedded rape myth that positions male bodies as inherently unrapeable, rendering the victim's account implausible before it has even been formally recorded. That this challenge is delivered in Sepedi is also significant, as it signals that these attitudes are not merely procedural but are rooted in culturally and linguistically specific constructions of Black masculinity that equate manhood with sexual invulnerability. Officers Jeanette, Mosidi and Dimpho confirmed the structural weight of stigma: The excerpts below provide accounts of why police officers think male victims of rape do not report.

“*It is still a stigma. It's just hard for men to come and report.”* (Jeanette, Police officer)

“*People still have that stigma.”* (Mosidi, Police officer)

“*There are some (men) who feel small when they report.”* (Dimpho, Police officer)

The repetition of the word “still” across Jeanette and Mosidi's responses is telling, suggesting an awareness that stigma persists despite broader social progress, and that these officers understand silence not as a personal failing of victims but as a rational response to an environment that has not yet made reporting safe. Dimpho's observation that men “feel small” when reporting is particularly significant, as it locates the harm not in the rape itself but in the act of disclosure, revealing how the police station can compound rather than alleviate the original trauma. Together, these accounts point to a form of anticipatory secondary victimization, where the fear of institutional judgment becomes as much of a barrier as the stigma itself. This aligns with findings from Naidoo (2013), who notes that male victims often face significant stigma and shame, which can deter reporting. Segopotso added by noting that male victims may:

“*Feel ashamed to go to the police station to stand right there in front of the policemen and women and say I have been raped by a woman. They feel somehow it takes away the power that they have as men.”* (Segopotso, Police officer)

Segopotso's account adds a further layer by introducing the specific scenario of female-perpetrated rape, which unsettles not only dominant constructions of masculinity but also the normative gender scripts that frame women as victims and men as perpetrators within South African rape discourse. The image of a man standing “in front of the policemen and women” to report being raped by a woman captures the vulnerability of that moment, where the victim must perform his victimhood before an audience whose institutional culture may be ill-equipped to receive it. It is also important to note that the reversal of expected roles may intensify feelings of shame and confusion for male victims The framing of rape as something that “takes away the power” men hold reflects how deeply sexual violence against men is experienced as an assault on social identity, not only on the body.

This reality is also reflected within the rural-urban geographical divide, Mosidi added:

“*In rural areas, people still have that stigma. So as a male saying that, a man from that family did that to me. First of all, they will be like, you are accusing the wrong person before it even reaches the police*”. (Mosidi, Police officer)

The emphasis on rural areas in this excerpt points to a significant disparity between urban and rural attitudes toward male rape. This aligns with research underlining that traditional beliefs and conservative attitudes are often more pronounced in rural South African communities (Jewkes et al., 2006, 2011). It is also worth noting that these rural communities are largely Black communities, and that the cultural scripts Mosidi describes are therefore racially situated. The stigma that prevents reporting is not simply a matter of geography or tradition in the abstract, but reflects how race, place, and patriarchal gender norms intersect to produce particular barriers for Black male victims of rape (Ratele, 2014; Gqola, 2015). Rural communities, more so than individuals in urban spaces, continue to hold onto traditional beliefs that stigmatize male rape victims. This stigma acts as a significant barrier to reporting and seeking justice (Sikweyiya and Jewkes, 2009). The phrase “a man from that family did that to me” suggests that many cases of male rape occur within family or community networks. This complicates reporting due to fears of disrupting family relationships and community harmony, which even previously have often been highly valued in rural South African contexts (Meursing et al., 1995).

Moreover, the statement above by Mosidi suggests an awareness within law enforcement of the cultural barriers they face in addressing male rape cases in rural areas. It highlights the need for culturally sensitive approaches to policing and community engagement (Faull, 2011). Mosidi's statement also highlights the need for targeted interventions that address not only the legal and procedural aspects of handling male rape cases but also the underlying cultural beliefs and community dynamics that often prevent these cases from even reaching the police. Thabo stated that rape cases are “unlike a person that has been robbed, this one is, it is quite a different one. It is private, and it needs a professional person.”

Thabang adds that, often, male victims will think:

“*What will other people do or say once they find out I have been turned into a woman? ‘Cause that's usually the perception they hold. You being raped is more like you being turned into a woman. I guess it's more to do with pride”*. (Thabang, Police officer)

The statement by Thabang reflects the pervasive belief in the South African society that associates victimhood, particularly in cases of sexual violence, with femininity. The idea of a man being “turned into a woman” through rape highlights the deeply rooted gender stereotypes and the perceived loss of masculinity associated with being a victim of sexual assault. The fear also points to the prevalent homophobia in South African society. The concern about what “other people do or say” highlights the fear of social repercussions faced by male rape victims.

The interviews provided further examples of these victim-blaming myths. Dimpho noted that male victims may not come forward due to fears that they will be seen as ‘weak' and ‘not man enough', explaining: “There are some (men) who feel small when they report.” Fani agreed that men's reluctance stems from a sense of emasculation, stating “they do not bring it to the authorities” because “they are ashamed.” This equation of victimhood with failed masculinity was a recurrent subtheme. Eugene described a case where a male victim disclosed being raped by his wife but had difficulty voicing it. Eugene stated, “When the wife gets back from work and the guy is maybe tired, then it becomes an issue of who are you sleeping with if you are not sleeping with me.” Eugene had to explain to him “that as a man you can be raped, your wife can rape you. As long as I don't consent, and then she forces me, that one is a rape victim.”‘This account illustrates how dominant constructions of masculinity position men as always sexually willing and in control within heterosexual relationships, making it difficult for male victims to recognize or articulate experiences of sexual coercion by female partners as rape. Eugene's need to explain that non-consensual sex within marriage constitutes rape highlights how patriarchal and heteronormative beliefs can obscure male victimhood and reinforce the idea that victimization represents a failure of masculinity.

### 5.2 Heteronormativity, homophobia, and the policing of male victimhood

Although SAPS officers receive training on handling rape cases, their responses to male rape victims remain influenced by broader cultural and heteronormative beliefs, including persistent myths surrounding male victimization, discussed in the previous section.

As Dimpho noted:

“*Men feel sorry if they open a case against a woman*.” (Dimpho, Police officer)

Dimpho's quote reveals several intersecting social dynamics. Firstly, opening a case against a woman may be seen as a violation of traditional masculine norms, potentially leading to feelings of guilt and shame (Walfield et al., 2024). At the same time, patriarchal constructions of women as inherently vulnerable or in need of protection may make it difficult for some men to position women as perpetrators of sexual violence. Secondly, the lack of robust support systems for male victims may contribute to feelings of isolation and regret when reporting (Naidoo, 2013). Lastly, some men may lack awareness of their legal rights or how rape is defined, further complicating their willingness to report victimization.

Still on the topic of male rape myths and within the broader heteronormative conceptualization of gender and sexuality, Abel stated the following:

“*Even if you agree that let's have sex. But, that “back part” is not used for sex, it's rape”*. (Abel, Police officer)

This excerpt by Abel reflects deeply ingrained cultural and social attitudes that significantly impact how male rape is perceived and addressed in the South African context. The statement implies that anal penetration, even if consensual, is not considered a legitimate form of sexual activity. This attitude can lead to the dismissal or minimization of male rape cases, particularly those involving anal penetration (Sikweyiya and Jewkes, 2009). The quote further suggests a problematic understanding of consent. It implies that certain sexual acts are inherently wrong or criminal, regardless of consent, which can complicate investigations and legal proceedings in male rape cases.

Moreover, the comment reflects a heteronormative view of sexuality that does not acknowledge the diverse sexual practices of LGBTQA+ individuals. This bias can result in inadequate responses to male rape cases, especially those involving same-sex assaults (Kiss et al., 2020; Javaid, 2018). In many South African cultures, discussions about anal sex remain taboo. This cultural silence can contribute to a lack of awareness and understanding about male rape, making it difficult for victims to report and for the police to respond appropriately (Ratele, 2014). Thabo in his interview stated that SAPS members:

“*Don't take mercy when victims come to report. How do you ask a man, where did they put it, in front or at the back*.” (Thabo, Police officer)

This quote reflects the stigma surrounding male sexual victimization within policing contexts. Thabo's reaction suggests a severe lack of empathy and compassion toward male rape victims. This attitude can significantly deter victims from reporting and seeking justice (Jewkes and Abrahams, 2002). The crude nature of the question further demonstrates a lack of professionalism and sensitivity in handling male rape cases. Such questioning can be profoundly traumatizing for victims and may constitute secondary victimization (Vetten, 2011).

When participants were asked whether they have dealt with male victims of rape, Stephen noted,

*I have dealt with one, yes. The only thing is that, from the onset, it was disguised from me. It was a gay person. So, he was having a penis and breasts. So, from the onset, I knew to be dealing with a woman. Only up until I started taking down the details and saw the ID number, and saw that it is a male ID number and even the names. If I remember, the name was X*^1^ *and that's when it raised suspicions, and I asked what is happening and then I asked him, let me say her. I asked her, how do you have this name as X, and from seeing what the ID number is, the seventh number after your birth date is over four, and which in my knowledge is a male ID number and then he said, yes it has something to do with Home Affairs, they are still sorting that out. And afterwards I said, okay it's fine, it is more personal. I continued with my work. Then I called the unit that deals with rape, the FCS (Family Violence, Child Protection and Sexual Offenses), then the person who came was a lady. Usually, if it is a lady coming in then they must, if it is a male victim driving with the female officer at night going to the hospital to go take samples and all that we usually give them another male to go with them just for safety so when we assumed it was a lady, the lady was okay to go with her but when they got to the hospital and now he has to undress they found it is male, so the lady came back and complained at me and said why do you let me drive with a male person alone then I said no the person to me she looked like a woman. So, but to answer your question, uh if you are professional, it doesn't matter who comes to you. If it is a male or whether a female saying I have been raped, we treat them the same*. (Stephen, Police officer)

Stephen's initial confusion about the victim's gender highlights the challenges police face when encountering transgender or gender non-conforming individuals. This aligns with findings from (Müller 2018), who in their study noted the invisibility and annihilation of queer bodies often leading to potential misunderstandings and inadequate responses.

The discrepancy between the victims' appearance and their official identification documents points to broader issues within South African bureaucracy regarding gender recognition. The process of changing gender markers on official documents can be lengthy and complex, often leaving transgender individuals with documentation that does not match their gender identity (Swarr, 2012; Spade, 2007). Stephen's approach to handling the sensitive information about the victim's gender identity demonstrates a level of professionalism. However, it also raises questions about privacy and the need for clear protocols when dealing with transgender victims.

The practice of assigning same-gender officers to accompany victims for medical examinations reflects an attempt at sensitivity. However, as this case demonstrates, such policies may not always account for the complexities of gender identity. This aligns with research by (Rumney 2008a,b), who argues that rigid gender-based policies in handling rape cases can sometimes create unintended challenges, particularly for male and LGBTQA+ victims.

Interestingly Segopotso noted with concern that:

“*For some other gay men, I don't know if it's an achievement or what. I don't know. Like, I fail to understand them because I would say maybe I know a few friends of mine who have normalized like they think it's ok. They think it's an achievement*.” (Segopotso, Police officer)

Segopotso's suggestion that some gay men” may perceive such experiences as an “achievement” reflects a problematic understanding of male rape and reveals troubling assumptions about sexual violence, particularly within the LGBTQ+ community. The quote further highlights internalized homophobia within some segments of the gay community, where violent or non-consensual sexual experiences are misconstrued as validating one's sexual identity (Msibi, 2012; Cannon, 2005). In the South African context, where homophobia is still prevalent in many areas, some gay individuals might internalize the idea that any sexual attention, even violence, is preferable to rejection or invisibility (Muthien, 2013). This view also reflects a coping mechanism within a marginalized community, where reframing traumatic experiences as positive is a misguided attempt at resilience (Cock, 2003; Scheepers, 2023). It is crucial to note that this view is not representative of the entire gay community in South Africa and is a harmful misconception and that the data of this study underlined that some police officers actively resisted homophobic attitudes. Segopotso and Tebogo, for instance, advocated for the SAPS to be educated about LGBTQI issues so that the police may be welcoming and not homophobic. Segopotso specifically emphasized the need for “sensitivity training on masculinities and sexualities” to ensure that “if you are deployed with someone gay, you know how to treat them professionally.”

Segopotso, in this study, mentions two incidents of discrimination whilst working for two stations. In the first incident, he mentions that while with his other colleagues, an older police officer passed a comment that, “I don't know what to call him”, and whilst starting at the new station, the station commander referred to him as “her”; he then turned around to seek clarity, and that is when the station commander said, “I don't know if you prefer to be called a him or her”. This frustrated him because he did not disclose his sexuality to the station commander. In the interview, Segopotso advocated for the SAPS to do more regarding issues of the LGBTIQ+ community by stating, “SAPS can do more when it comes to addressing LGBTQ issues”. These accounts show that sexuality matters in the workplace.

Moreover, Segopotso's advocacy for gender-sensitive training, specifically to improve professional interactions with gay individuals, reflects a growing awareness of the need for more inclusive and equitable policing practices in South Africa. This perspective is particularly significant given the country's complex history with LGBTQ+ rights and the ongoing challenges faced by this community (Müller, 2018). The statement implicitly acknowledges existing deficiencies in how some police officers interact with gay individuals. This aligns with research indicating that LGBTQ+ people in South Africa often face discrimination and insensitive treatment from law enforcement (Abaver and Cishe, 2018; Naidoo, 2013).

While not yet the norm, these calls for greater inclusion signal a shift away from the heteronormative, victim-blaming cultures that have traditionally silenced male victims within the SAPS. The call for training suggests a recognition that these issues stem, at least partially, from a lack of understanding and education rather than inherent prejudice. By addressing these gaps through comprehensive training programs, the police can develop a more nuanced understanding of male vulnerability and the various contexts in which male rape occurs.

The authors also identified the intersection of male rape stigma with homophobia and heteronormative masculinity ideals in the data. Segopotso asserts:

“*Most of the time, they (police officers) think gay people are liars. They are attention seekers because they just seek attention. You know, so it is like when you're a gay person, you walk into a police station and then you say, I've been raped. And this is what happened. Then in their minds, they're like, oh, here comes another attention seeker, this particular person is seeking attention*.”

Segopotso's statement reveals a disturbing perception of how police officers may view gay male rape victims, highlighting a significant barrier to justice and support for this vulnerable population. The belief that gay individuals reporting rape are “attention seekers” reflects a harmful intersection of homophobia and rape myths within the police. This attitude not only dismisses the legitimate trauma experienced by gay male victims but also perpetuates a cycle of underreporting and inadequate investigation of these crimes (Rumney, 2009). Such preconceived notions among police officers can lead to secondary victimization, where the victim's experience is invalidated by the very system meant to protect them, potentially exacerbating the psychological impact of the assault and deterring future victims from coming forward (Turchik and Edwards, 2012). In the interview, Segopotso recounted a case where a gay male victim was “turned away” and told to “come back when he was sober”, a clear instance of secondary victimization based on sexual orientation.

The officer's account of a male rape victim being “sent away and told to come back when he was sober” reveals several troubling issues in law enforcement's approach to male rape cases in South Africa. Firstly, this response demonstrates a lack of understanding of the complex relationship between alcohol consumption and sexual victimization. Research highlights that perpetrators often use alcohol to facilitate sexual assault (Jewkes and Abrahams, 2002). By dismissing the victim due to intoxication, the police may be inadvertently protecting the perpetrator and reinforcing harmful myths about rape (Machisa et al., 2011). Secondly, this action reflects a broader societal tendency to doubt or discredit male rape victims, particularly when substance use is involved (Sikweyiya and Jewkes, 2009). In the South African context, where traditional masculinity norms are strong, male victims already face significant barriers to reporting. Being turned away by the police likely compounds their trauma and discourages future reporting (Dworkin et al., 2017).

Pockets of resistance to these victim-blaming myths also emerged. Dimpho maintained that officers should take victims' statements without judgment,'as it is not their role to determine whether they are right or wrong. Similarly, Tebogo advocated for visible victim-friendly messaging at every station that explicitly welcomes vulnerable groups, including LGBTI individuals, and affirms their right to report cases like any person. He further stressed that homophobic attitudes are incompatible within the responsibilities of policing. This resistance reflects a growing recognition that addressing personal biases is crucial for maintaining public trust and ensuring equitable treatment for all victims regardless of their gender and sexual orientation [or identity] (Alderden and Ullman, 2012). This resistance suggests that some police officers are challenging cultural scripts that doubt and blame male victims of rape.

## 6 Discussion

In exploring how rape myths, stigma, and sociocultural beliefs shape officers' understandings of male victimization, the findings revealed the enduring power of stereotypes that compromise adequate responses. Participants' accounts of male rape being viewed as emasculating, doubted as possible, or associated with homosexuality align with patterns documented across multiple contexts. From the United States (Turchik and Edwards, 2012) to Australia (Lowe and Balfour, 2015) and the United Kingdom (Rumney, 2008a,b), police have similarly been found to trivialize or disbelieve male victims due to masculine norms that cast men as invulnerable. These narratives echo Weiss's (2010) application of Connell's theory; whereby male victims are perceived as falling short of hegemonic masculine ideals and are therefore relegated to a subordinated masculinity deemed less credible.

This discrediting intensifies further at the intersections of masculinity and marginalized sexualities, with participants confirming that gay male victims experience a “double stigma” in which homophobic biases compound rape myths (Javaid, 2015a; Rumney, 2008a,b). Segopotso, for instance, noted that male rape is often interpreted as evidence that the victim is gay, highlighting how assumptions about sexuality shape perceptions of victimhood. These findings resonate with research showing that queer identities in South Africa have historically been marginalized through colonial-era sodomy laws and apartheid constructions of patriarchal, heteronormative masculinity (Semugoma et al., 2012; Shaw, 2018). Research further suggests that men who have sex with men face heightened risks of sexual violence while remaining reluctant to disclose abuse due to stigma and fear of ridicule (Lane et al., 2008). Tebogo and Segopotso similarly observed that gay men may avoid reporting rape because they anticipate homophobic treatment from police officials.

While these patterns of male rape myths within law enforcement are well established in Western contexts, this study contributes by centring perspectives from the Global South. South African officers' reflections demonstrate how such beliefs are embedded within local constructions of masculinity. Participant descriptions of male victims being shamed for failing to uphold ideals of aggression and toughness echo findings from Suriname, where police questioned how a “real man” could be raped (Bailey and Desai, 2011; Barrow, 2019). Likewise, cultural proverbs such as “monna ke nku ha a lle” [a man is a sheep, he does not cry] reveal how stoicism and emotional restraint are embedded within local gender norms. Although the expectation that “real men” must remain emotionally invulnerable appears across cultures, these locally specific idioms demonstrate how masculine scripts become encoded within distinct socio-linguistic contexts (Fahlberg and Pepper, 2016; Govinda, 2020; Marhia, 2012; Nilan et al., 2008). By surfacing these Indigenous expressions, the study illuminates cultural nuances that more universalising Western theories may overlook (Moller, 2007). Crucially, these expressions are not only gendered but racialised. The proverb “monna ke nku ha a lle” circulates within specific Black South African linguistic communities whose constructions of masculinity were shaped in part by the disruptions of colonialism and apartheid, which simultaneously undermined African men's social authority and demanded their performance of a particular kind of toughened stoicism (Morrell et al., 2012). Understanding how race shapes these gender scripts is therefore essential to understanding why male victimhood remains so difficult to articulate within these communities, and why Black male victims face a particular set of racialised and gendered barriers when they approach the SAPS.

The findings also suggest that institutional police culture reinforces these stereotypes. Shared laughter among officers, for example, appeared to normalize the dismissal of male rape victims and function as a bonding mechanism that reinforces group cohesion at victims' expense (Faull, 2011). Such interactions may simultaneously silence victims and deter future reporting by signaling that male rape allegations are not taken seriously (Karlsson and Evaldsson, 2011). At the same time, this laughter may reflect discomfort and inadequate training among officers dealing with male rape cases (Marks, 2005).

Plummer's (1995, 1996) concept of “telling sexual stories” further assists in understanding why heterosexual men may be reluctant to report rape. Because sexual storytelling is shaped by hierarchies of power, domination, and marginalization, heterosexual male victims may fear that disclosure will undermine their masculine status, heterosexual identity, or intimate relationships. Remaining silent therefore becomes a strategy for preserving social legitimacy and avoiding ridicule, including sexist or homophobic responses from police officials. In tightly knit South African communities, fears of ostracism and shame may further discourage disclosure and help-seeking (Abrahams and Gevers, 2017).

Participants also highlighted how police questioning itself may retraumatise male victims. Similar findings emerged in Widanaralalage et al.'s (2022) study, where participants described police questioning as emotionally “bludgeoning” because it forced victims to recount graphic details of abuse. Such findings point to the need for more trauma-informed policing practices. Comparatively, Gregory and Lees (1999) found that both male and female rape victims often perceived police officers, particularly male officers, as unsympathetic and ill-equipped to deal sensitively with sexual violence cases. This suggests that dismissive responses may stem not only from rape myths but also from broader inadequacies in police training and understandings of sexual violence.

## 7 Conclusion

This research provides important insights into the institutional barriers and biases that male victims encounter when seeking support and justice from the SAPS. The study highlights the experiences and perspectives of SAPS officers, who shared their insights through in-depth interviews. It reveals how prevailing notions of masculinity, heteronormativity, and organizational culture often silence and invalidate the experiences of male victims of rape.

The findings underscore the need for initiatives aimed at improving access for male victims to the police, to address and challenge the myths and stereotypes surrounding male rape. Additionally, this study not only questions existing biases but also encourages officers to confront their perceptions of masculinity, sexuality, and victimhood through facilitated discussions and interactive training.

The study contributes to the global literature by foregrounding how these dynamics are mediated within a Global South context, where locally specific constructions of masculinity, language, and cultural norms shape the intelligibility of male victimhood in distinct ways. By drawing attention to indigenous expressions, sociocultural expectations, and the intersection of gender with sexuality and social location, the paper demonstrates that hegemonic masculinity operates through contextually embedded meanings rather than as a uniform global construct. This not only extends intersectional analyses of victimization but also challenges the dominance of Western frameworks in the study of policing and rape, offering a more nuanced account of how institutional power, culture, and identity intersect to structure access to justice.

## Data Availability

The raw data supporting the conclusions of this article will be made available by the authors, without undue reservation.

## Footnotes

1 The name X is used to protect the identity of the victim.
